# Crystal structure of (*E*)-*N*-[(2-meth­oxy­naphthalen-1-yl)methyl­idene]-3-nitro­aniline

**DOI:** 10.1107/S2056989015020502

**Published:** 2015-11-14

**Authors:** Devika Bhai R., C. R. Girija, Shalini Suresh, Ramakrishna Reddy

**Affiliations:** a Research & Development Centre, Bharathiar University, Coimbatore 641 046, India; b Govt. Science College, Nrupathunga Road, Bangalore 560 001, India; c SSMRV College, Jayanagar 4th T block, Bangalore 560 041, India

**Keywords:** crystal structure, naphthaldimine Schiff base, hydrogen bonding

## Abstract

In the title compound, C_18_H_14_N_2_O_3_, the dihedral angle between the naphthalene ring system and the benzene ring is 59.99 (13)°. A short intra­molecular C—H⋯N contact closes an *S*(6) ring. The nitro group is disordered over two orientations in a statistical ratio. In the crystal, weak C—H⋯O hydrogen bonds and very weak π–π stacking inter­actions [centroid–centroid separation = 3.9168 (17) Å] are observed.

## Related literature   

For background to Schiff bases, see: Tolulope *et al.* (2013 ▸).
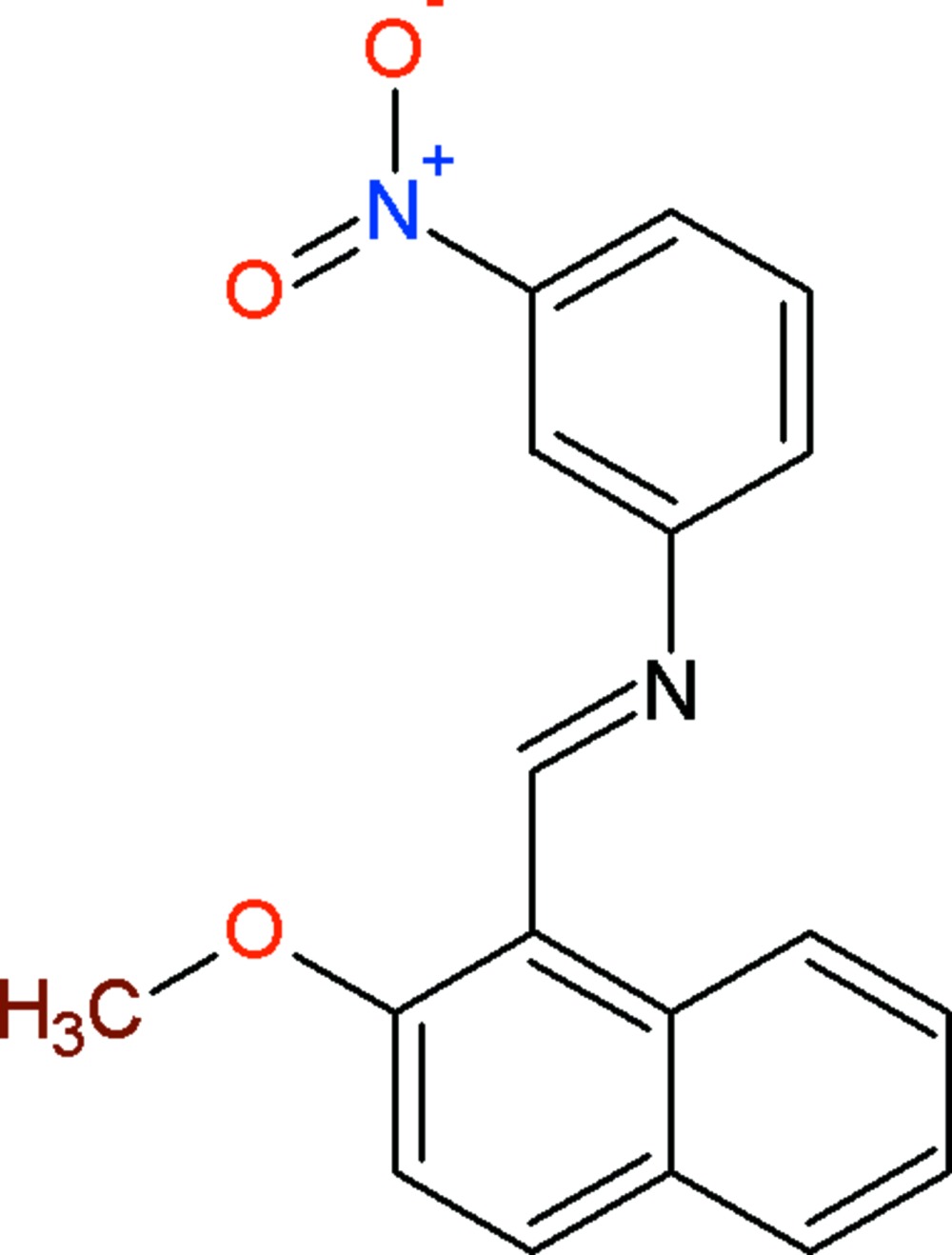


## Experimental   

### Crystal data  

C_18_H_14_N_2_O_3_
*M* *_r_* = 306.31
Monoclinic, 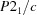

*a* = 12.8481 (7) Å
*b* = 15.4085 (6) Å
*c* = 7.6232 (3) Å
β = 98.040 (4)°
*V* = 1494.33 (12) Å^3^
*Z* = 4
Mo *K*α radiation
μ = 0.09 mm^−1^
*T* = 293 K
0.35 × 0.30 × 0.25 mm

### Data collection  

Bruker Kappa APEXII CCD diffractometer
Absorption correction: multi-scan (*SADABS*; Bruker, 2004 ▸) *T*_min_ = 0.957, *T*_max_ = 0.989
21149 measured reflections
2622 independent reflections
1646 reflections with *I* > 2σ(*I*)
*R*_int_ = 0.046

### Refinement  

*R*[*F* ^2^ > 2σ(*F* ^2^)] = 0.055
*wR*(*F* ^2^) = 0.143
*S* = 1.14
2622 reflections
227 parameters
42 restraints
H-atom parameters constrained
Δρ_max_ = 0.16 e Å^−3^
Δρ_min_ = −0.17 e Å^−3^

### 

Data collection: *APEX2* (Bruker, 2004 ▸); cell refinement: *APEX2* and *SAINT* (Bruker, 2004 ▸); data reduction: *SAINT* and *XPREP* (Bruker, 2004 ▸); program(s) used to solve structure: *SIR92* (Altomare *et al.*, 1993 ▸); program(s) used to refine structure: *SHELXL2014* (Sheldrick, 2015 ▸); molecular graphics: *OLEX2* (Dolomanov *et al.*, 2009 ▸); software used to prepare material for publication: *SHELXL2014*.

## Supplementary Material

Crystal structure: contains datablock(s) I. DOI: 10.1107/S2056989015020502/hb7529sup1.cif

Structure factors: contains datablock(s) I. DOI: 10.1107/S2056989015020502/hb7529Isup2.hkl

Supporting information file. DOI: 10.1107/S2056989015020502/hb7529Isup3.cml

**Fig. 1.**
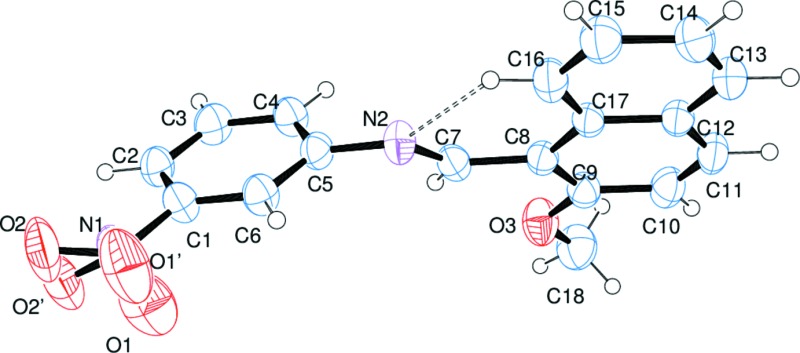
Plot of the title compound showing the intramolecular C—H···N interaction as a dashed line.

. DOI: 10.1107/S2056989015020502/hb7529fig1.tif
Plot of the title compound showing the intra­molecular C—H⋯N inter­action as a dashed line.

**Fig. 2.**
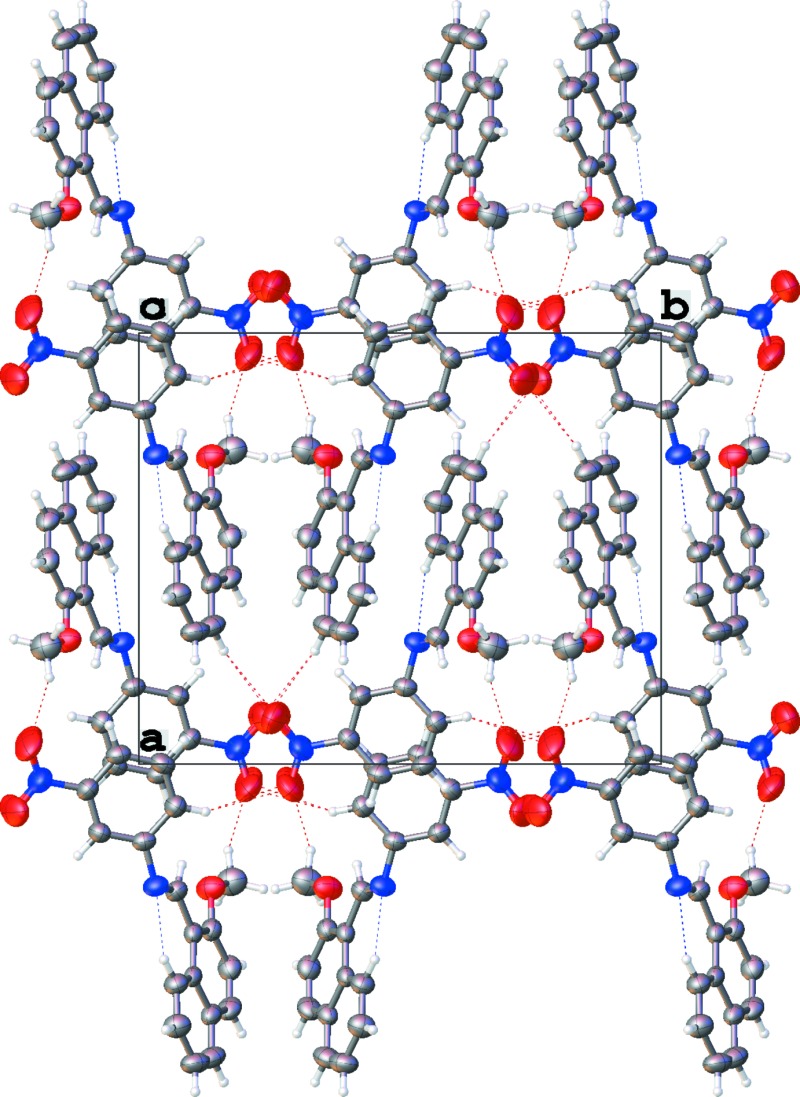
Crystal packing diagram showing the C—H···N and C—H···O interactions as dashed lines

. DOI: 10.1107/S2056989015020502/hb7529fig2.tif
Crystal packing diagram showing the C—H⋯N and C—H⋯O inter­actions as dashed lines

CCDC reference: 1429914

Additional supporting information:  crystallographic information; 3D view; checkCIF report

## Figures and Tables

**Table 1:** Hydrogen-bond geometry (Å, °)

*D*—H⋯*A*	*D*—H	H⋯*A*	*D*⋯*A*	*D*—H⋯*A*
C16—H16⋯N2	0.93	2.31	2.961 (3)	127
C13—H13⋯O1′^i^	0.93	2.49	3.318 (14)	148
C18—H18*A*⋯O2^ii^	0.96	2.46	3.135 (18)	127

Symmetry codes: (i) 
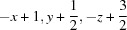
; (ii) 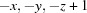.

## supplementary crystallographic information

### S1. Chemical context

Schiff bases are considered an important class of organic compounds, which have wide applications. In recent years, they have gained significant inter­est in the area of drug research and development owing to the broad bioactivities such as insecticidal, anti­bacterial, anti­tuberculosis and anti­microbial reported for the compounds and their metal complexes. These compounds play an important role in biological systems and are observed in various enzymes such as transaminases, tryptophan synthase etc. The important physical and biological properties of these compounds are related to the presence of the intra­molecular hydrogen bond and proton transfer equilibrium. Schiff bases have also been utilized as ligands to synthesize metal complexes with inter­esting applications. The steric and inductive effects introduced by substituents present on the aromatic portion of the Schiff base can influence the properties of the ligand significantly. In continuation of our efforts in understanding the role of subtle electronic variations such as substituent effects on Chemistry and activity of Schiff bases and their metal complexes, we herein report the crystal structure of Schiff base derived from 3-nitro­aniline and 2-meth­oxy naphthaldehyde (Tolulope *et al.*, 2013).

### S2. Structural commentary

The molecule of title compound is non-planar, with a dihedral angle between the naphthyl and phenyl aromatic rings of 59.99 (13)°, in which the two rings are twisted from one another. The C9—O3 single bond of 1.358 (3) Å and the C7═N2 double bond of 1.261 (3) Å .The bond angle of C5—N2—C7 of the imine group is 117.5 (2)°, less than 120°. The bond length of the nitro group is N1—O2(1.241 (6)Å) and N1—O1(1.239 (6)Å) and bond angle in O2—N1—O1 is 123.4 (12)° which is more than the planar bond angle of 120°. The torsion angles C8—C7—N2—C5 is 178.1 (2)°.These values support that the configurations about the N2=C7 bond is anti­(E-form), which is in accordance with the enol-imine tautomeric form.

### S3. Supra­molecular features

The title compound has an intra molecular C16— H16···N2 hydrogen bond forming an S(6) motif (Table 2). Also there is a C—H···O inter­molecular inter­action, in which a C—H of the naphthyl ring of one molecule and O-atom of the nitro group of another molecule are linked to one another.

### S4. Synthesis and crystallization

The block-like, yellow single crystals of the compound C_18_ H_14_ N_2_ O_3_, were grown using 1:1 mixture of CHCl_3_ and methanol as solvent by slow evaporation technique.

### S5. Refinement

The hydrogen atoms in the structure were positioned geometrically (C—H = 0.93–0.98 Å. N—H = 0.86Å) and were refined using a riding model with Uiso(H) = xUeq(C,N), where x = 1.5 for methyl and 1.2 for all other atoms. The two oxygen atoms of the nitro group are disordered over two orientations. The SADI, SIMU, and ISOR commands in SHELXL (Sheldrick, 2015) were used to model the disorder.

Crystal data, data collection and structure refinement details are summarized in Table 1.

### Crystal data

**Table:**

C_18_H_14_N_2_O_3_	*D*_x_ = 1.362 Mg m^−^^3^
*M**_r_* = 306.31	Melting point: 407 K
Monoclinic, *P*2_1_/*c*	Mo *K*α radiation, λ = 0.71073 Å
*a* = 12.8481 (7) Å	Cell parameters from 5867 reflections
*b* = 15.4085 (6) Å	θ = 2.6–29.9°
*c* = 7.6232 (3) Å	µ = 0.09 mm^−^^1^
β = 98.040 (4)°	*T* = 293 K
*V* = 1494.33 (12) Å^3^	Block, yellow
*Z* = 4	0.35 × 0.30 × 0.25 mm
*F*(000) = 640	

### Data collection

**Table:**

Bruker Kappa APEXII CCD diffractometer	2622 independent reflections
Radiation source: fine-focus sealed tube	1646 reflections with *I* > 2σ(*I*)
Graphite monochromator	*R*_int_ = 0.046
ω and φ scan	θ_max_ = 25.0°, θ_min_ = 2.1°
Absorption correction: multi-scan (*SADABS*; Bruker, 2004)	*h* = −15→15
*T*_min_ = 0.957, *T*_max_ = 0.989	*k* = −18→18
21149 measured reflections	*l* = −9→8

### Refinement

**Table:**

Refinement on *F*^2^	42 restraints
Least-squares matrix: full	Hydrogen site location: inferred from neighbouring sites
*R*[*F*^2^ > 2σ(*F*^2^)] = 0.055	H-atom parameters constrained
*wR*(*F*^2^) = 0.143	*w* = 1/[σ^2^(*F*_o_^2^) + (0.0403*P*)^2^ + 0.9181*P*] where *P* = (*F*_o_^2^ + 2*F*_c_^2^)/3
*S* = 1.14	(Δ/σ)_max_ < 0.001
2622 reflections	Δρ_max_ = 0.16 e Å^−^^3^
227 parameters	Δρ_min_ = −0.17 e Å^−^^3^

### Special details

**Table:**

Geometry. All e.s.d.'s (except the e.s.d. in the dihedral angle between two l.s. planes) are estimated using the full covariance matrix. The cell e.s.d.'s are taken into account individually in the estimation of e.s.d.'s in distances, angles and torsion angles; correlations between e.s.d.'s in cell parameters are only used when they are defined by crystal symmetry. An approximate (isotropic) treatment of cell e.s.d.'s is used for estimating e.s.d.'s involving l.s. planes.

### Fractional atomic coordinates and isotropic or equivalent isotropic displacement parameters (Å^2^)

**Table:**

	*x*	*y*	*z*	*U*_iso_*/*U*_eq_	Occ. (<1)
C1	0.0584 (2)	−0.09762 (18)	0.7746 (4)	0.0523 (7)	
C2	−0.0226 (2)	−0.03905 (19)	0.7675 (4)	0.0523 (7)	
H2	−0.0893	−0.0562	0.7877	0.063*	
C3	−0.0015 (2)	0.0457 (2)	0.7296 (4)	0.0566 (8)	
H3	−0.0545	0.0870	0.7247	0.068*	
C4	0.0976 (2)	0.07014 (18)	0.6986 (4)	0.0517 (7)	
H4	0.1103	0.1276	0.6710	0.062*	
C5	0.17862 (19)	0.01015 (18)	0.7081 (3)	0.0461 (7)	
C6	0.1585 (2)	−0.07494 (18)	0.7487 (4)	0.0498 (7)	
H6	0.2118	−0.1162	0.7583	0.060*	
C7	0.2942 (2)	0.07229 (17)	0.5418 (4)	0.0475 (7)	
H7	0.2333	0.0823	0.4630	0.057*	
C8	0.39198 (19)	0.10445 (16)	0.4875 (3)	0.0430 (6)	
C9	0.3839 (2)	0.13912 (17)	0.3178 (4)	0.0485 (7)	
C10	0.4724 (2)	0.17407 (18)	0.2520 (4)	0.0571 (8)	
H10	0.4658	0.1972	0.1383	0.069*	
C11	0.5665 (2)	0.17361 (19)	0.3552 (4)	0.0585 (8)	
H11	0.6242	0.1970	0.3107	0.070*	
C12	0.5808 (2)	0.13905 (17)	0.5278 (4)	0.0480 (7)	
C13	0.6798 (2)	0.1397 (2)	0.6330 (4)	0.0622 (8)	
H13	0.7372	0.1626	0.5870	0.075*	
C14	0.6933 (2)	0.1077 (2)	0.7988 (5)	0.0649 (9)	
H14	0.7595	0.1082	0.8662	0.078*	
C15	0.6069 (2)	0.0736 (2)	0.8696 (4)	0.0622 (8)	
H15	0.6161	0.0521	0.9847	0.075*	
C16	0.5094 (2)	0.07154 (18)	0.7716 (4)	0.0537 (7)	
H16	0.4532	0.0484	0.8211	0.064*	
C17	0.49240 (19)	0.10383 (16)	0.5969 (3)	0.0424 (6)	
C18	0.2726 (3)	0.1775 (2)	0.0501 (4)	0.0753 (10)	
H18A	0.2006	0.1711	−0.0023	0.113*	
H18B	0.2897	0.2381	0.0620	0.113*	
H18C	0.3176	0.1502	−0.0242	0.113*	
N1	0.0376 (2)	−0.18893 (19)	0.8121 (5)	0.0849 (9)	
N2	0.28245 (17)	0.03270 (16)	0.6825 (3)	0.0544 (6)	
O3	0.28735 (15)	0.13796 (14)	0.2188 (3)	0.0669 (6)	
O1	0.1053 (10)	−0.2414 (9)	0.778 (4)	0.106 (5)	0.50 (4)
O2	−0.0433 (13)	−0.2105 (10)	0.869 (4)	0.108 (5)	0.50 (4)
O1'	0.1105 (8)	−0.2356 (11)	0.876 (4)	0.106 (5)	0.50 (4)
O2'	−0.0556 (6)	−0.2107 (9)	0.772 (3)	0.097 (4)	0.50 (4)

### Atomic displacement parameters (Å^2^)

**Table:**

	*U*^11^	*U*^22^	*U*^33^	*U*^12^	*U*^13^	*U*^23^
C1	0.0460 (16)	0.0463 (17)	0.0646 (19)	−0.0062 (13)	0.0072 (14)	0.0025 (14)
C2	0.0372 (15)	0.061 (2)	0.0598 (18)	−0.0039 (13)	0.0102 (13)	−0.0005 (15)
C3	0.0424 (16)	0.059 (2)	0.069 (2)	0.0107 (14)	0.0099 (14)	0.0020 (15)
C4	0.0476 (16)	0.0463 (16)	0.0620 (18)	−0.0013 (13)	0.0096 (13)	0.0044 (14)
C5	0.0354 (14)	0.0553 (18)	0.0477 (16)	−0.0035 (13)	0.0059 (12)	−0.0008 (13)
C6	0.0393 (15)	0.0476 (17)	0.0625 (18)	0.0038 (12)	0.0077 (13)	0.0042 (13)
C7	0.0418 (15)	0.0458 (16)	0.0548 (18)	−0.0055 (12)	0.0063 (13)	−0.0008 (13)
C8	0.0444 (16)	0.0331 (14)	0.0543 (17)	−0.0011 (11)	0.0163 (13)	−0.0015 (12)
C9	0.0487 (17)	0.0414 (16)	0.0577 (18)	−0.0013 (12)	0.0149 (14)	−0.0002 (13)
C10	0.062 (2)	0.0522 (18)	0.0618 (19)	−0.0004 (14)	0.0238 (16)	0.0072 (14)
C11	0.0534 (19)	0.0560 (19)	0.072 (2)	−0.0088 (14)	0.0310 (16)	−0.0021 (15)
C12	0.0446 (16)	0.0420 (16)	0.0609 (18)	−0.0049 (12)	0.0196 (14)	−0.0074 (13)
C13	0.0451 (18)	0.072 (2)	0.073 (2)	−0.0127 (15)	0.0206 (16)	−0.0146 (17)
C14	0.0406 (17)	0.081 (2)	0.073 (2)	−0.0059 (15)	0.0070 (15)	−0.0106 (18)
C15	0.0538 (19)	0.073 (2)	0.0598 (19)	−0.0040 (16)	0.0084 (15)	0.0028 (16)
C16	0.0436 (16)	0.0553 (18)	0.0644 (19)	−0.0043 (13)	0.0146 (14)	0.0028 (15)
C17	0.0420 (15)	0.0313 (14)	0.0567 (17)	−0.0024 (11)	0.0164 (13)	−0.0051 (12)
C18	0.074 (2)	0.091 (3)	0.061 (2)	−0.0056 (19)	0.0080 (17)	0.0119 (18)
N1	0.062 (2)	0.0590 (19)	0.136 (3)	−0.0098 (16)	0.021 (2)	0.0112 (19)
N2	0.0412 (13)	0.0627 (16)	0.0610 (15)	−0.0060 (11)	0.0132 (11)	0.0063 (13)
O3	0.0566 (13)	0.0810 (15)	0.0629 (13)	−0.0057 (11)	0.0072 (10)	0.0200 (11)
O1	0.086 (5)	0.061 (4)	0.170 (12)	0.014 (3)	0.012 (6)	0.019 (6)
O2	0.094 (6)	0.071 (4)	0.172 (12)	−0.023 (4)	0.066 (6)	0.021 (8)
O1'	0.083 (5)	0.069 (5)	0.165 (12)	0.003 (4)	0.008 (6)	0.056 (7)
O2'	0.074 (4)	0.067 (4)	0.153 (11)	−0.025 (3)	0.029 (4)	0.002 (7)

### Geometric parameters (Å, º)

**Table:**

C1—C2	1.372 (4)	C11—H11	0.9300
C1—C6	1.374 (4)	C11—C12	1.407 (4)
C1—N1	1.468 (4)	C12—C13	1.406 (4)
C2—H2	0.9300	C12—C17	1.425 (3)
C2—C3	1.372 (4)	C13—H13	0.9300
C3—H3	0.9300	C13—C14	1.345 (4)
C3—C4	1.380 (4)	C14—H14	0.9300
C4—H4	0.9300	C14—C15	1.402 (4)
C4—C5	1.386 (4)	C15—H15	0.9300
C5—C6	1.380 (4)	C15—C16	1.366 (4)
C5—N2	1.418 (3)	C16—H16	0.9300
C6—H6	0.9300	C16—C17	1.410 (4)
C7—H7	0.9300	C18—H18A	0.9600
C7—C8	1.463 (3)	C18—H18B	0.9600
C7—N2	1.261 (3)	C18—H18C	0.9600
C8—C9	1.390 (4)	C18—O3	1.412 (3)
C8—C17	1.435 (4)	N1—O1	1.241 (6)
C9—C10	1.412 (4)	N1—O2	1.227 (6)
C9—O3	1.358 (3)	N1—O1'	1.227 (6)
C10—H10	0.9300	N1—O2'	1.239 (6)
C10—C11	1.347 (4)		
			
C2—C1—C6	123.1 (3)	C11—C12—C17	118.9 (3)
C2—C1—N1	118.7 (3)	C13—C12—C11	121.4 (3)
C6—C1—N1	118.2 (3)	C13—C12—C17	119.7 (3)
C1—C2—H2	121.2	C12—C13—H13	119.3
C3—C2—C1	117.6 (3)	C14—C13—C12	121.4 (3)
C3—C2—H2	121.2	C14—C13—H13	119.3
C2—C3—H3	119.7	C13—C14—H14	120.2
C2—C3—C4	120.7 (3)	C13—C14—C15	119.6 (3)
C4—C3—H3	119.7	C15—C14—H14	120.2
C3—C4—H4	119.6	C14—C15—H15	119.6
C3—C4—C5	120.9 (3)	C16—C15—C14	120.9 (3)
C5—C4—H4	119.6	C16—C15—H15	119.6
C4—C5—N2	122.9 (2)	C15—C16—H16	119.4
C6—C5—C4	118.8 (2)	C15—C16—C17	121.1 (3)
C6—C5—N2	118.2 (2)	C17—C16—H16	119.4
C1—C6—C5	118.9 (2)	C12—C17—C8	118.8 (2)
C1—C6—H6	120.6	C16—C17—C8	124.0 (2)
C5—C6—H6	120.6	C16—C17—C12	117.2 (2)
C8—C7—H7	116.1	H18A—C18—H18B	109.5
N2—C7—H7	116.1	H18A—C18—H18C	109.5
N2—C7—C8	127.9 (3)	H18B—C18—H18C	109.5
C9—C8—C7	116.0 (2)	O3—C18—H18A	109.5
C9—C8—C17	119.2 (2)	O3—C18—H18B	109.5
C17—C8—C7	124.8 (2)	O3—C18—H18C	109.5
C8—C9—C10	121.2 (3)	O1—N1—C1	115.6 (9)
O3—C9—C8	117.1 (2)	O2—N1—C1	121.0 (8)
O3—C9—C10	121.7 (3)	O2—N1—O1	123.4 (12)
C9—C10—H10	120.3	O1'—N1—C1	119.3 (8)
C11—C10—C9	119.5 (3)	O1'—N1—O2'	126.6 (12)
C11—C10—H10	120.3	O2'—N1—C1	114.1 (8)
C10—C11—H11	118.7	C7—N2—C5	117.5 (2)
C10—C11—C12	122.5 (3)	C9—O3—C18	119.7 (2)
C12—C11—H11	118.7		
			
C1—C2—C3—C4	0.5 (4)	C9—C8—C17—C16	179.0 (2)
C2—C1—C6—C5	−2.1 (4)	C9—C10—C11—C12	0.3 (4)
C2—C1—N1—O1	164.6 (15)	C10—C9—O3—C18	−4.2 (4)
C2—C1—N1—O2	−13.9 (19)	C10—C11—C12—C13	−179.8 (3)
C2—C1—N1—O1'	−156.0 (17)	C10—C11—C12—C17	−0.6 (4)
C2—C1—N1—O2'	24.5 (14)	C11—C12—C13—C14	179.1 (3)
C2—C3—C4—C5	−1.1 (4)	C11—C12—C17—C8	0.3 (4)
C3—C4—C5—C6	0.2 (4)	C11—C12—C17—C16	−178.6 (2)
C3—C4—C5—N2	−178.1 (2)	C12—C13—C14—C15	−0.5 (5)
C4—C5—C6—C1	1.3 (4)	C13—C12—C17—C8	179.6 (2)
C4—C5—N2—C7	−53.8 (4)	C13—C12—C17—C16	0.6 (4)
C6—C1—C2—C3	1.1 (4)	C13—C14—C15—C16	0.7 (5)
C6—C1—N1—O1	−15.6 (15)	C14—C15—C16—C17	−0.2 (5)
C6—C1—N1—O2	165.9 (18)	C15—C16—C17—C8	−179.3 (3)
C6—C1—N1—O1'	23.9 (17)	C15—C16—C17—C12	−0.4 (4)
C6—C1—N1—O2'	−155.6 (13)	C17—C8—C9—C10	−0.4 (4)
C6—C5—N2—C7	127.9 (3)	C17—C8—C9—O3	179.7 (2)
C7—C8—C9—C10	178.5 (2)	C17—C12—C13—C14	−0.1 (4)
C7—C8—C9—O3	−1.4 (3)	N1—C1—C2—C3	−179.0 (3)
C7—C8—C17—C12	−178.7 (2)	N1—C1—C6—C5	178.1 (3)
C7—C8—C17—C16	0.2 (4)	N2—C5—C6—C1	179.7 (2)
C8—C7—N2—C5	178.1 (2)	N2—C7—C8—C9	174.4 (3)
C8—C9—C10—C11	0.2 (4)	N2—C7—C8—C17	−6.8 (4)
C8—C9—O3—C18	175.7 (3)	O3—C9—C10—C11	−179.9 (3)
C9—C8—C17—C12	0.1 (4)		

### Hydrogen-bond geometry (Å, º)

**Table:**

*D*—H···*A*	*D*—H	H···*A*	*D*···*A*	*D*—H···*A*
C16—H16···N2	0.93	2.31	2.961 (3)	127
C13—H13···O1′^i^	0.93	2.49	3.318 (14)	148
C18—H18*A*···O2^ii^	0.96	2.46	3.135 (18)	127

Symmetry codes: (i) −*x*+1, *y*+1/2, −*z*+3/2; (ii) −*x*, −*y*, −*z*+1.
