# Supplementary figures and images for: Crystal structure of (E)-N-[(2-meth­oxy­naphthalen-1-yl)methyl­idene]-3-nitro­aniline
Source: Acta Crystallogr E Crystallogr Commun. 2015 Nov 14;71(Pt 12):o941–2. doi: 10.1107/S2056989015020502 (PMC4719912; doi:10.1107/S2056989015020502)

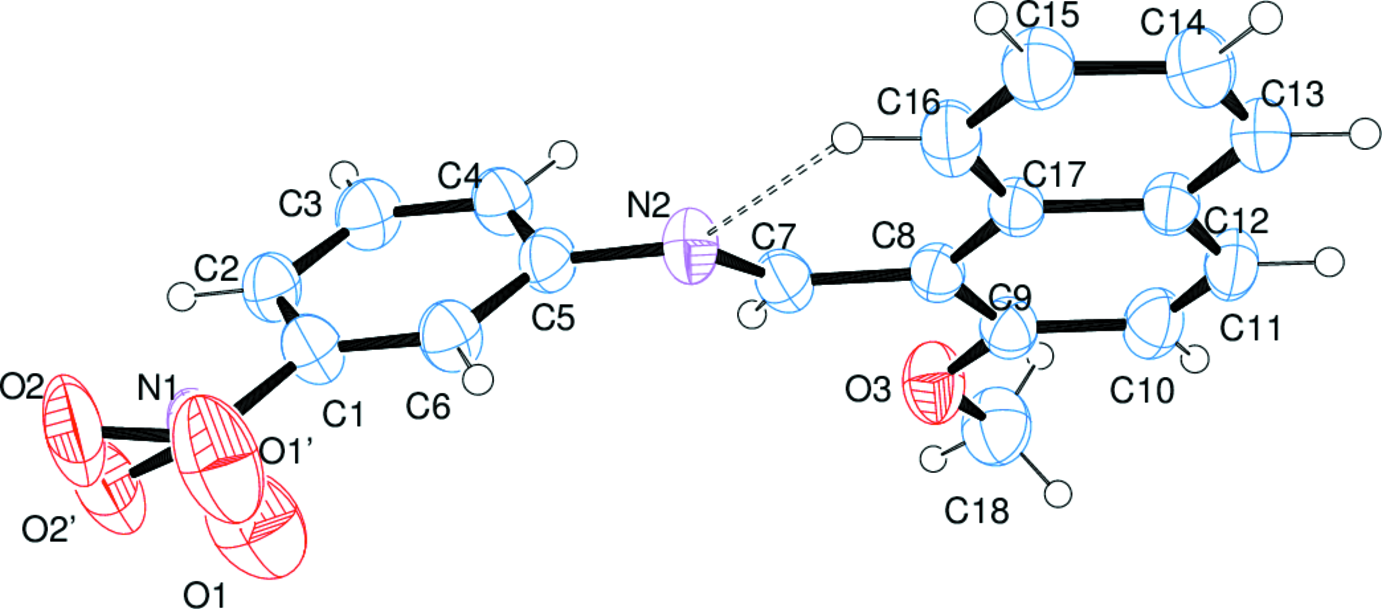

Supplement: Supplementary file 4 [file e-71-0o941-fig1.tif]

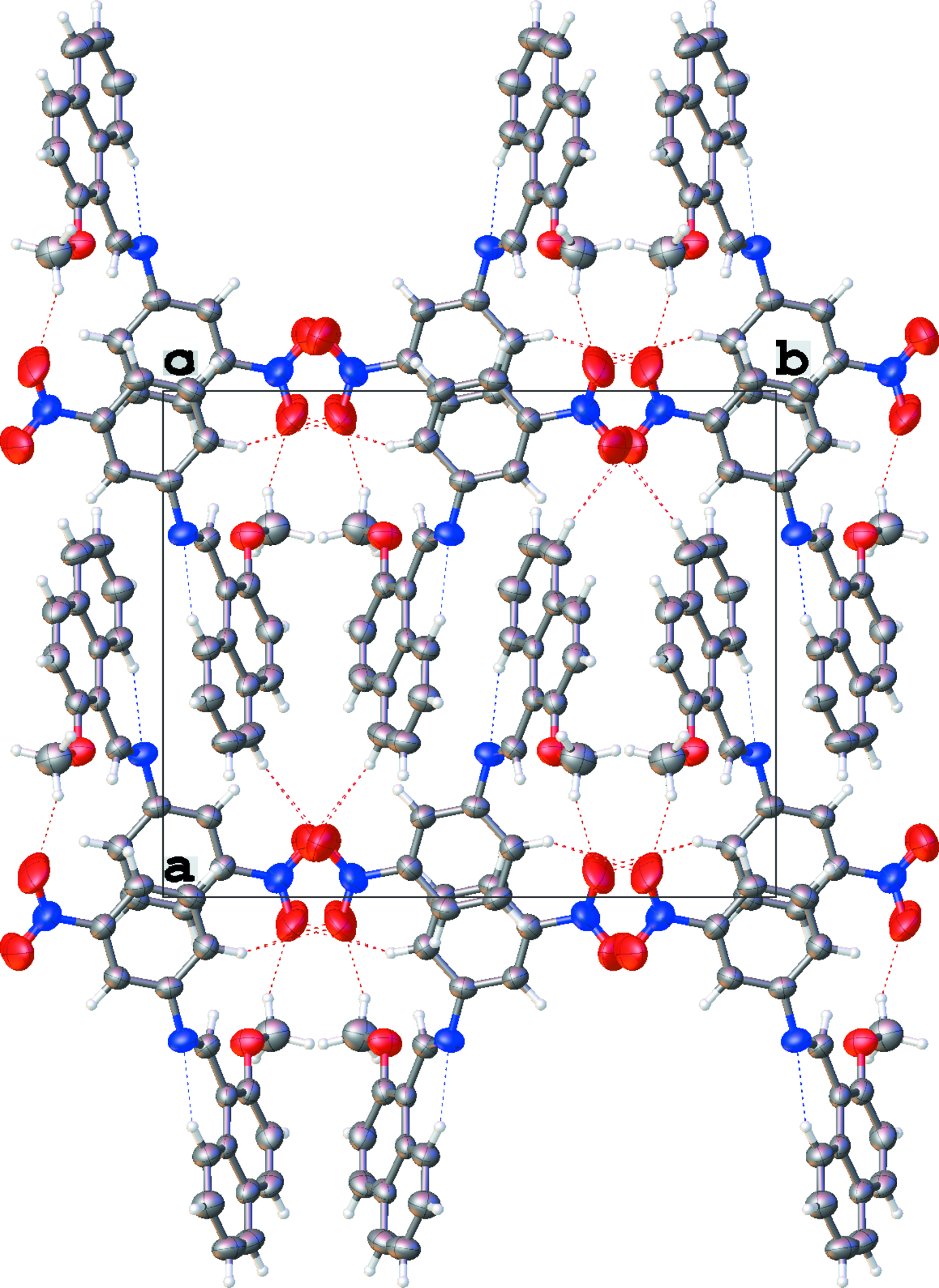

Supplement: Supplementary file 5 [file e-71-0o941-fig2.tif]
